# P-2182. HPV Genotyping Prevalence and Risk Factors Among Women from Two Rural Communities of Peru

**DOI:** 10.1093/ofid/ofaf695.2345

**Published:** 2026-01-11

**Authors:** Lauren Jernstadt, Ruben K Briceno, Neysa Miller, Olivia B Slewa, Giovanna Russano, Elizabeth Lossada-Soto, Coral Chen

**Affiliations:** Michigan State University, East Lansing, Michigan; Michigan State University, East Lansing, Michigan; Michigan State University, East Lansing, Michigan; Michigan State University, East Lansing, Michigan; Michigan State University, East Lansing, Michigan; Michigan State University, East Lansing, Michigan; Michigan State University, East Lansing, Michigan

## Abstract

**Background:**

Cervical cancer is the second leading cause of death among Peruvian women, and remains a challenge for the Peruvian health care system. Although the vaccination program coverage in Peru is growing, it’s still insufficient, showing disparities in health care access. Also, the vaccine Gardasil 4 may not be sufficient to cover the different Human Papillomaviruses (HPV) of the Peruvian population.

**Figure:**
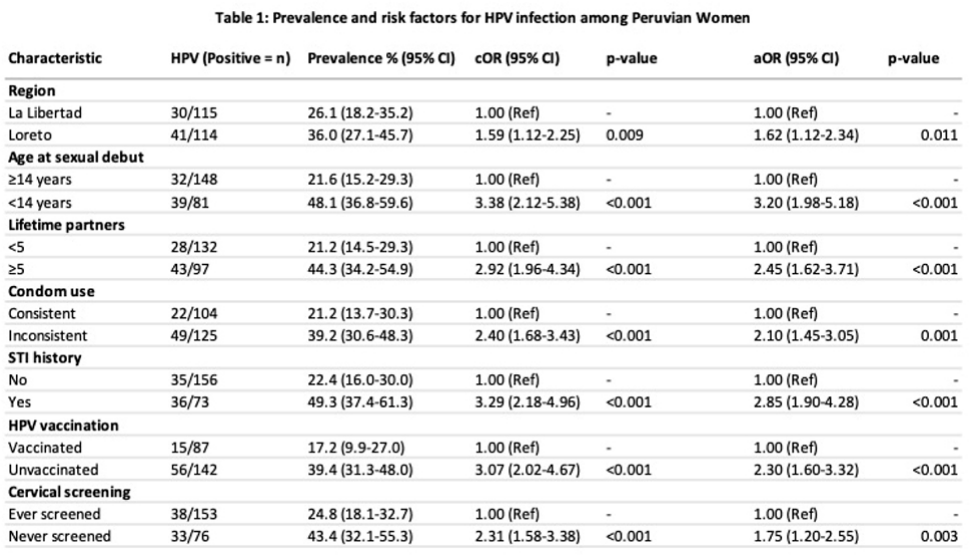

**Methods:**

A cross-sectional study was conducted in 2024. We collected cervical cell samples from La Libertad and Loreto, with different ethnicities in two different regions. A total of 229 (La Libertad: 115, Loreto: 114) women between the ages of 13 and 60 years undergoing cervical screening were enrolled in the study. All samples were analyzed by polymerase chain reaction, and HPV presence and genotyping were obtained. Demographic, sexual history, and risk factors data were collected using a validated questionnaire. Multivariate logistic regression was performed for the statistical analysis.

**Results:**

HPV overall prevalence was 31.0%. Loreto 36.0%; La Libertad 26.1% (p=0.012). The High Risk (HR) genotypes 16, 18, and 45 were found in 34%, 22%, and 18%, respectively. Early onset of sexual intercourse has a strong prediction for HPV infection. (95% CI 1.98-5.18, p< 0.001), STI diagnosis (aOR=2.85, 95% CI: 1.90-4.28). consistency of condom use (aOR= 2.10, 95% CI: 1.45-3.05).

**Conclusion:**

HPV genotypes and prevalence were very high in our study compared to the national statistics in Peru. Loreto has a high incidence (31%) compared to La Libertad (26.1%) and appears to follow the prevalence trend observed in North America, with HPV type 16 accounting for cases. In regression analysis, early sexual intercourse onset (< 14 years) was the most significant risk factor for HPV infection. Our findings evidence the need for a national program with targeted screening in high-risk population.

**Disclosures:**

All Authors: No reported disclosures

